# Pharmacokinetic, Pharmacogenetic, and Other Factors Influencing CNS Penetration of Antiretrovirals

**DOI:** 10.1155/2016/2587094

**Published:** 2016-09-29

**Authors:** Jacinta Nwamaka Nwogu, Qing Ma, Chinedum Peace Babalola, Waheed Adeola Adedeji, Gene D. Morse, Babafemi Taiwo

**Affiliations:** ^1^Department of Pharmaceutical Chemistry, Faculty of Pharmacy, University of Ibadan, Ibadan, Nigeria; ^2^Centre for Drug Discovery Development and Production, Faculty of Pharmacy, University of Ibadan, Ibadan, Nigeria; ^3^School of Pharmacy and Pharmaceutical Sciences and New York State Center of Excellence in Bioinformatics and Life Sciences, University at Buffalo, 701 Ellicott Street, Buffalo, NY 14203, USA; ^4^Pharmacy Practice (Medicine and Pediatrics), School of Pharmacy and Pharmaceutical Sciences, University at Buffalo, 285 Kapoor Hall, Buffalo, NY 14214, USA; ^5^Center for Integrated Global Biomedical Sciences, University at Buffalo, Buffalo, NY 14203, USA; ^6^Department of Clinical Pharmacology, University College Hospital, Ibadan, Nigeria; ^7^Division of Infectious Diseases, Center for Global Health, Feinberg School of Medicine, Northwestern University, 645 N. Michigan Avenue, Suite 900, Chicago, IL, USA

## Abstract

Neurological complications associated with the human immunodeficiency virus (HIV) are a matter of great concern. While antiretroviral (ARV) drugs are the cornerstone of HIV treatment and typically produce neurological benefit, some ARV drugs have limited CNS penetration while others have been associated with neurotoxicity. CNS penetration is a function of several factors including sieving role of blood-brain and blood-CSF barriers and activity of innate drug transporters. Other factors are related to pharmacokinetics and pharmacogenetics of the specific ARV agent or mediated by drug interactions, local inflammation, and blood flow. In this review, we provide an overview of the various factors influencing CNS penetration of ARV drugs with an emphasis on those commonly used in sub-Saharan Africa. We also summarize some key associations between ARV drug penetration, CNS efficacy, and neurotoxicity.

## 1. Introduction

Human immunodeficiency virus (HIV) remains a leading cause of morbidity and mortality, especially in sub-Saharan Africa where 70% of the people living with HIV globally live. HIV penetrates the central nervous system (CNS) within a few days of infection [1–4], establishing residence in macrophages and microglia cells and producing CNS inflammation that may lead to neuronal injury and neurological complications. Combination antiretroviral therapy (ART) for HIV effectively suppresses plasma HIV viremia [5–7] and as a result considerably increases life expectancy [8]. ART also confers neurological benefit in most individuals by suppressing CNS viral replication and inflammation. However, up to 40% of individuals exhibit neurocognitive impairment despite successful suppression of plasma viremia [9]. Potential explanations for this include poor penetration of ARV drugs into the CNS, which may allow continued HIV replication and inflammation in that compartment [10]. In addition, some antiretroviral drugs may be neurotoxic. In this review article, we provide an overview of the various factors influencing the CNS penetration of antiretroviral drugs. These include general factors such as drug transporters, the blood-brain barrier, and blood-cerebrospinal fluid barrier and host specific factors that are driven by pharmacokinetics and pharmacogenetics. Other factors include physicochemical properties of the antiretroviral drug, local cerebral blood flow, and chronic inflammation. We also summarize associations between antiretroviral drug penetrations, CNS efficacy, and neurotoxicity.

## 2. Data Collection Methods

We conducted a comprehensive query of PubMed and Google Scholar. Search terms used included pharmacogenetics, Africa, antiretrovirals, zidovudine, efavirenz, tenofovir, saquinavir, raltegravir, enfuvirtide, bevirimat, nevirapine, ritonavir, maraviroc, zalcitabine, delavirdine, amprenavir, indinavir, didanosine, nelfinavir, lopinavir, stavudine, atazanavir, fosamprenavir, abacavir, tipranavir, emtricitabine, darunavir, lamivudine, Central Nervous System, blood flow, penetrat^*∗*^ , HIV, blood brain barrier, CSF, CSF concentration, transporters, P-gp, ABCB1, protein binding, plasma concentration, and drug interaction. Additional references were obtained from the reference lists in the articles identified using this search method. Only articles published in English language were reviewed.

## 3. Commonly Used Antiretroviral Drugs in Sub-Saharan Africa

The World Health Organization (WHO) recommends that the first-line ART should consist of a nonnucleoside reverse transcriptase inhibitor (NNRTI) and two nucleos(t)ide reverse transcriptase inhibitors (NRTIs), one of which should be zidovudine (ZDV) or tenofovir disoproxil fumarate (TDF). Efavirenz (EFV) is the preferred NNRTI in ART regimens in sub-Saharan Africa [11, 12], although some patients are treated with nevirapine- (NVP-) based ART [13–15]. Other NRTIs commonly used as first-line treatment are lamivudine (3TC) and emtricitabine (FTC). Both didanosine (ddI) and stavudine (d4T) are rarely used these days because of their toxicities [16, 17]. The WHO recommendation for second-line ART consists of a ritonavir-boosted protease inhibitor (PI) plus two or three NRTIs, one of which should be ZDV or TDF, depending on what was used in first-line therapy. Atazanavir with ritonavir (ATV/r) and lopinavir/ritonavir (LPV/r) are the preferred PIs. Saquinavir (SQV) has fallen out of favor partly because it has a high pill-burden, while indinavir (IDV) has a high risk of toxicity, and fosamprenavir (FPV) is relatively expensive. Although the integrase strand transfer inhibitor, raltegravir, is an option for second-line therapy when combined with a boosted PI, integrase inhibitors are typically reserved for third-line regimens. Other components of third-line therapy include drugs likely to have anti-HIV activity such as the second-generation NNRTI etravirine and the boosted PI darunavir/ritonavir (DRV/r), both of which are rarely available due to relatively high costs [18]. Dolutegravir (DTG), a newer integrase strand transfer inhibitor, is not yet available in sub-Saharan Africa but has the potential to gain attention in this region in the next few years due to its attractive safety, efficacy, and resistance profile. DTG was demonstrated to be superior in first-line ART to EFV [19, 20] and DRV/r [21] in large randomized trials.

## 4. CNS Penetration of Different Antiretroviral Drugs

Penetration of antiretrovirals into the CNS is critical to optimize suppression of the CSF HIV viral load and overall replication in the CNS. It has been suggested that the use of antiretroviral compounds with poor penetration into the CNS may be associated with an increased risk of cognitive decline. This is however controversial as shown by the proposed CNS Penetration-Effectiveness (CPE) ranking system, which categorizes antiretrovirals into three groups (low, intermediate, and high CNS penetration) [10] or four groups [22] based on their chemical properties, concentration in the CSF, and effectiveness in reducing the CSF viral load. Studies evaluating associations between CPE score and neurocognitive outcomes have produced inconsistent results [23–25], although lower CPE ranking correlated with higher HIV viral loads in CSF [10]. Studies on CNS penetration of commonly used antiretrovirals and their CPE rankings are shown in Table 1. Of note, the same antiretroviral drug displays variations in CSF concentration (varying penetrating abilities) in different patients within the same study and in different studies. This likely reflects the multifactorial determinants of CNS drug penetration.

## 5. Factors Affecting CNS Penetration of Antiretroviral Drugs

### 5.1. General Factors

#### 5.1.1. Blood-Brain Barrier and Blood-CSF Barrier

The blood-brain barrier (BBB) and blood-CSF barrier (BCSFB) are normal anatomical structures that evolved to protect the CNS from toxic substances. In HIV-infected individuals, however, these barriers also limit penetration and, potentially, efficacy of some antiretrovirals in the brain. The BBB is formed by brain capillary endothelial cells fused together by tight junctions, hence characterized by lack of fenestration and the paucity of pinocytosis [26, 27]. On the other hand, BCSFB, which separates CSF and blood, consists of the choroid plexus and the arachnoid membrane. The choroid plexus epithelium is involved in numerous exchange processes that increase the CSF concentrations of nutrients and hormones and decrease the CSF concentrations of potentially deleterious compounds and metabolites [28]. These characteristics restrict the penetration of large or hydrophilic molecules through the BBB and BCSFB, selectively permitting penetration of small and lipophilic molecules. Thus, antiretrovirals such as NVP, ZDV, EFV, and FTC, with physicochemical properties that support penetration, have an advantage in penetrating the BBB by simple diffusion. Any condition which compromises the BBB and/or BCSFB will likely increase the rate of entrance of drugs into the brain [29].

#### 5.1.2. Drug Transporters

Transporters are membrane proteins that facilitate the movement of molecules into or out of cells. They can be categorized in different ways including efflux and influx transporters or the adenosine triphosphate binding cassette (ABC) and solute carrier (SLC) transporters. The CNS penetration of antiretroviral drugs that are substrates of drug transporters is partly dependent on the level of expression of the transporters. Due to the substrate specificity of these transporters, drugs that possess significant similarities to them are transported into or out of cells. Efflux and influx transporters can play a critical role in determining drug concentrations in the systemic circulation and in cells. However, the overall rate constant for efflux of drug from the brain is approximately 75-fold higher and from CSF is 8-fold higher than the respective rate constants for influx [30]. This implies that efflux of drugs out of cells occurs more frequently when compared to drug influx into the cells, due to ubiquitous expression of efflux transporters. The emphasis is on the ABC transporters, for example, permeability-glycoprotein (P-gp) and multidrug resistance-associated proteins (MRPs), because of their effect on the CNS penetration of some antiretrovirals.

The brain penetration of antiretrovirals that are substrates of transport proteins is partly affected by these transporters. The efflux pumps (P-gp, MRPs, and breast cancer resistance protein (BCRP)) are present at the BBB and have been shown to extrude substrate drugs from this site. Thus, they limit PIs from penetrating the CNS [31–36] due to their affinity for these transporters. The most important efflux transporter influencing the brain penetration of antiretrovirals is P-gp, because of its multiple binding sites for substrates and inhibitors [37, 38]. Apart from PIs, P-gp limits brain penetration of the NRTIs, abacavir, and ZDV [32, 39] and can also efflux some structurally unrelated hydrophobic molecules.

The expression and functionality of P-gp can be modulated by inhibition and induction, which can affect the pharmacokinetics, efficacy, safety, or tissue levels of P-gp substrates [40]. For example, PIs (substrates of P-gp) generally have poor brain penetration, but their penetrability may be enhanced by coadministration with specific P-gp inhibitor such as ritonavir [36]. Consistent with this, a study in nonhuman primates reported that P-gp inhibition at the BBB significantly enhanced the distribution of nelfinavir into the brain [41]. A previous study showed that patients with HIV encephalitis have higher brain P-gp levels compared to patients without HIV encephalitis [42], suggesting that patients with HIV encephalitis may be predisposed to lower CNS penetration of substrate drugs. In addition, MRP has been reported to contribute to the poor brain penetrations of PIs [43].

### 5.2. Host Specific Factors

#### 5.2.1. Pharmacogenetics

Pharmacogenetics is the discipline that analyses the genetic basis for the interindividual variation in the body disposition of drugs [44]. Pharmacogenetics has found application in the treatment of numerous diseases including HIV infection. Since pharmacogenetics can predict drug exposure, hence response to therapy or risk of toxicity, it is of particular importance for the drugs that have a narrow therapeutic index and/or metabolic pathways affected by polymorphisms in the drug metabolizing enzymes.

*Cytochrome P450 2B6* has received much attention in HIV therapy due to its ubiquitous role in the metabolism of antiretroviral drugs.* CYP2B6* is highly polymorphic [45, 46] and is characterized by wide interindividual variability in expression and activity [47]. Both EFV and NVP are mainly metabolized by* CYP2B6* with African populations having higher poor metabolizer frequency [48, 49], hence potentially prone to development of adverse reactions with these agents. Indeed, previous studies have reported significant associations of some* CYP2B6* variants with elevated plasma EFV [50–55], which is relevant to CNS EFV levels/effects since higher plasma concentration may result in higher CNS penetration. In line with this, Winston and Puls in their study, though with a small sample size, reported an association of CSF EFV concentration with* CYP2B6* genotype [56]. Further,* CYP2B6* polymorphism also affects NVP plasma levels [57–59]. NVP concentrations increased by 92% with the presence of* CYP2B6 516T* allele and decreased by 31% with the presence of *CYP*3*A*5^*∗*^3 in Malawians [60]. However, another study reported that* CYP2B6 516/983* genotypes had no effect on NVP concentrations [61].  Table 2 shows the wide variability in poor metabolizer frequencies in different African populations as reported in different studies. This observation suggests that results from one African population should not be extrapolated to other African populations, since Africans are very heterogenic with respect to drug disposition and pharmacogenetics. This is in line with recommended multinational clinical trial across sub-Saharan Africa in order to validate the EFV dose recommendation [53].

In addition, polymorphisms in drug transporter genes can influence penetration of substrate drugs into the CNS. Illustratively, polymorphism in* ABCB1* was shown to influence plasma concentrations of NFV [62] and of EFV [63, 64]. It was also reported that* ABCB1 c.3435C>T* contributed to NVP-induced hepatotoxicity risk [14]. On the contrary, CSF RAL concentrations did not differ by* ABCB1 3435C>T* genotype in healthy volunteers [65]. Significant variability in* ABCB1* genes has been reported in black South Africans [66]. Hence, antiretroviral CNS penetration may vary in such population.

The evidences to date suggest that genetic profile could be put into consideration prior to initiation of a given antiretroviral agent, especially those for which their primary metabolism is by enzyme(s) with genetic polymorphism. The most relevant drug in this respect in sub-Saharan Africa is EFV. However, much work still needs to be done to translate the potential of EFV pharmacogenetics into clinical practice. Other common antiretroviral drugs used in sub-Saharan Africa which are also affected by pharmacogenetics have been highlighted in Table 3.

*Pharmacogenetics and Efavirenz.* Despite the efficacy of EFV in viral suppression, neuropsychiatric side effects are common [57, 67–72], and some patients on EFV-based therapy discontinue treatment as a result of neurotoxicity and other adverse effects [67, 73, 74]. Some of these cases are possibly associated with* CYP2B6* polymorphisms that predispose to higher drug concentrations [75]. Accordingly, it has been suggested that a lower dose of EFV should be given to patients with poor metabolizer genotype compared to fast metabolizers with functional* CYP2B6* alleles [53, 76]. Of note, patients carrying *CYP*2*B*6^*∗*^6/^*∗*^18 showed extremely high plasma EFV concentrations compared to those carrying either *CYP*2*B*6^*∗*^1/^*∗*^1 or *CYP*2*B*6^*∗*^6/^*∗*^6, and *CYP*2*B*6^*∗*^6/^*∗*^6 patients also had higher plasma EFV concentrations than patients with *CYP*2*B*6^*∗*^1/^*∗*^1 genotype [77]. In another study *CYP*2*B*6^*∗*^6/^*∗*^16 was again associated with increased plasma EFV concentration [52]. These results showed that efavirenz plasma concentration may partly depend on* CYP2B6* genotype.

*CYP2A6* polymorphism* (CYP2A6 248T>G)* has also been reported to be associated with high EFV plasma level in a Ghanaian cohort study [78]. Cytochrome* P450 2A6* has minor contribution to the metabolism of EFV [79]. However, this pathway may become increasingly important for individuals with poor metabolizer* CYP2B6* genes. Dual* CYP2B6* and* CYP2A6* slow metabolism may lead to extremely high EFV exposure [79].

While it has been proposed that pharmacogenetic testing to identify patients carrying poor metabolizer genotypes may help optimize EFV dosing and minimize potential neurotoxicity from high EFV concentration, routine pharmacogenetic testing is not currently recommended. However, the finding that EFV 400 mg once daily dose is virologically noninferior and better tolerated than current 600 mg dosing [80] should motivate consideration of the lower dose for routine use.

### 5.3. Pharmacokinetics and Drug Specific Factors

#### 5.3.1. Drug Characteristics

*(a) Molecular Weight and Lipophilicity*. The physicochemical properties of antiretroviral drugs influence their entry into the CNS. Many drugs cross cellular membranes by simple diffusion, in which drug molecules diffuse freely across membrane from the area where the concentration is high to the area of lower concentration. The rate of penetration of a drug into the brain by simple diffusion depends on its lipid solubility and size [81]. Antiretrovirals with very high molecular weight tend to have relatively poor CNS penetration. For example, enfuvirtide, a fusion inhibitor with molecular mass above 4,000 Da, penetrates poorly into the CNS [82]. On the other hand, abacavir and ZDV with molecular weights of 286.332 g/mol and 267.242 g/mol, respectively, are better positioned to penetrate the CNS.

The lipophilic nature of the BBB preferentially allows penetration of low molecular weight molecules with optimal lipophilicity. Oil/water partition coefficient is a useful tool in predicting the lipid solubility of neutral molecules. The higher the partition coefficient, the greater the lipophilicity and the better the brain penetration of the drug. That means drugs with lower partition coefficient will not easily penetrate the BBB by simple diffusion. However, the optimal partition coefficient for good membrane penetration is about 100 [26]. Therefore, drugs with very high partition coefficients (1000) will also have lower diffusion capacity, because it is difficult for highly lipophilic drugs (lipid soluble) to diffuse from the lipid layer of the BBB into the brain extracellular fluid [26]. For acidic and basic drugs, the degree of ionization, which is pH dependent, determines lipid solubility. For example, weakly acidic drugs will exist in more unionized form at lower pH and the more a given drug exists in unionized form, the better the membrane permeability is. In contrast, weakly basic drugs will exist in more ionized form at lower pH. To illustrate the impact of this using Henderson-Hasselbalch equation, NVP which is a weakly basic drug with pKa of 2.8 [83] will exist largely in unionized form in plasma (pH of about 7.4) and this may be a contributing factor to good CNS penetration of NVP.

*(b) Protein Binding.* Drugs, especially lipophilic ones, bind to circulating proteins, which are usually albumin, acid glycoproteins, globulins, and lipoproteins. Different antiretroviral drugs vary in the extent to which they bind to protein. Only the unbound antiretroviral drugs are available to cross membrane. Nucleoside analogues like ZDV, 3TC, ddI, d4T, and abacavir have molecular weights less than 500 Da and low protein binding, allowing better CNS penetration. However, having a low molecular weight and low protein binding is not enough to ensure high CNS penetration. For example, tenofovir has low molecular weight (287.213 g/mol) and low protein binding and was reported to have low CNS penetration [84]. Contrary to most nucleoside analogues, PIs have high molecular weights (>500 Da) and protein binding (greater than 90%) with the exception of IDV [26, 85, 86] and are also substrates of efflux transporters. Molecular weight > 500 Da and protein binding > 90% generally impede membrane penetration of drugs [26], the characteristics that may contribute to the low brain penetration of PIs. Of note, many antiretrovirals exert substantial antiviral activity in the CNS even when the CSF concentration is low compared to plasma levels, provided the CSF levels exceed efficacy thresholds. This is the case with some boosted PIs such as DRV/r [87] and LPV/r [88]. Similarly, EFV is over 99% bound to protein [89] and has sufficient concentration in the CSF for viral suppression and induction of adverse events in that compartment. Generally, several factors combine to determine the CNS penetration and effects of different antiretroviral drugs. These factors have been summarized in Table 4.

#### 5.3.2. Drug Interaction

HIV infection is associated with several opportunistic infections [90, 91] as well as other coinfections and comorbidities. As a result, many HIV patients receiving ART also use concomitant medications for other conditions, thus predisposing to drug-drug interactions. Pharmacokinetic interactions occur when the precipitant drug (the drug causing the interaction) alters the concentrations of the object drug (affected drug). Many drug interactions occur as a result of enzyme induction or inhibition, which may lead to decrease or increase in the plasma concentration (and presumably CNS levels) of the object drug. The interactions are sometimes more complex with some drugs simultaneously inhibiting and inducing multiple enzymatic pathways or with two drugs exhibiting bidirectional interactions. Some drugs can also increase the brain uptake of other drugs from the blood through enzyme inhibition. For example, ketoconazole increased CSF concentration of ritonavir by 178% [92].

Tuberculosis is the most common opportunistic infection during HIV infection [93]. Several drug-drug interactions between antiretrovirals and antituberculosis agents have been reported, with the most dramatic ones occurring with rifampin, a potent* CYP 450* inducer. Illustratively, the plasma concentration of NVP was reduced by 37.3% [94] and the median AUC was reduced from 56.2 to 32.8 microg/mL per hour (−41.6%) when coadministered with rifampin [95]. This is mainly because rifampin induces the enzyme* CYP2B6*, which is responsible for the biotransformation of NVP. The interactions between rifampin and EFV are also substantial and may necessitate an increase in EFV dose when coadministered, while interactions between rifampin and boosted PIs are so consequential that coadministration is generally contraindicated [96–103]. Interactions may occur with other antituberculosis drugs; for example, isoniazid is a potent inhibitor of both* CYP2C19* and* CYP3A4* [104]. Therefore, concomitant administration of drugs that are substrate to both* CYP2C19* and* CYP3A4* like NFV, may lead to clinically important drug-drug interaction.

Importantly, several drug interactions that may facilitate CNS penetration of antiretrovirals occur via inhibition of the efflux transporters that are involved in limiting brain penetration of drugs. As such, CNS penetration of some antiretrovirals may be enhanced by coadministering suitable efflux inhibitor such as ritonavir [87], an inhibitor of efflux transporter and* CYP 450* enzymes [105]. Nicotine significantly increased SQV blood-to-brain transfer in rats through inhibition of efflux transporters [106]. Most clinically important drug-drug interactions can be explained in part by modulation of important transporters' activity.

Use of traditional medicine is a common practice, especially in Africa where patients often simultaneously seek treatment from both conventional and traditional health providers. The WHO estimated that up to 80% of the African population uses traditional medicine [107]. Some medicinal plants have been identified as having antiretroviral properties [91, 108, 109]. While the antiretroviral efficacy of such herbal products has not been demonstrated in well conducted randomized clinical trials, patients may resort to their use due to limited access to recommended antiretroviral drugs, intolerance to the conventional medicine, or cultural factors. Hence, herbal use is common among people on ART [110, 111]. Unfortunately, many HIV patients do not disclose this to their antiretroviral prescribers.

Many traditional medicines have complex metabolic pathways including CYP 450 enzymes [112] and may perpetrate clinically important drug interactions. For example, an in vitro study identified the potential for clinically significant drug interactions for both* H. hemerocallidea* and* Sutherlandia* (two African plants used for the treatment of HIV) through inhibition of* CYP3A4* and* P-glycoprotein* expression [113]. When these plants are taken together with antiretrovirals, this may lead to increased plasma concentration and CNS penetration of the substrate antiretroviral drugs. This may particularly enhance CNS penetration of PIs because they are substrates of both* CYP3A4* and* P-gp*. There is a paucity of data on the metabolism of medicinal plants in general. However, clinical studies and case reports involving many antiretroviral-herb pharmacokinetic interactions have been reviewed [114].

### 5.4. Other Factors

#### 5.4.1. Chronic Inflammation

The presence of chronic inflammation in HIV patients may compromise the BBB and affect the pattern of antiretroviral penetration into the CNS. In one study, 24.6% of patients treated with ART and 38.6% of untreated patients were found to have BBB alteration [115]. The percentage difference between treated and untreated individuals was not significant, suggesting that BBB impairment persists in some HIV patients even during ART. Consistent with this, other studies found persistent BBB impairment in some patients despite CSF viral load reduction after antiretroviral therapy [116, 117].

Alteration in the cells (e.g., pericytes, astrocytes, and endothelial cells) that provide support to the BBB can also affect CNS penetration of antiretrovirals, and this is common in HIV infection. Brain pericytes, for example, are positioned within the neurovascular unit to support BBB maintenance [118]. Brain pericyte coverage was found to be diminished in HIV-infected patients and this was associated with pericyte dysfunction in chronic neuroinflammation. These changes were accompanied by shrinking of tight junction protein and presence of phosphorylated occludin, indicative of BBB compromise [119]. Using a set of adult viable pericyte deficient mouse mutants, it was shown that pericyte deficiency increases the permeability of the BBB to water and a range of low molecular mass and high-molecular-mass tracers [120]. These data suggest enhanced CNS penetration of antiretrovirals in HIV positive individuals with persistent CNS inflammation compared to HIV negative individuals. On the contrary, in vitro experiments showed that chronic inflammation can upregulate* P-gp* expression and activity and so tighten the BBB to CNS-acting drugs that are P-gp substrates [121]. Therefore, the presence of chronic inflammation with subsequent disruption of BBB and the supporting cells in the brain may be an important determinant of CNS penetration of antiretroviral drugs.

#### 5.4.2. Local Cerebral Blood Flow

The brain receives 15–20% of the cardiac output, making it one of the most perfused organs in the body [122]. Factors that regulate the cerebral blood flow (CBF) include the net pressure gradient across the cerebral vascular beds (the most important of which is the mean arterial blood pressure) and the cerebral vascular resistance. These, together with the autoregulation process, allow the brain to control the cerebral blood perfusion [123]. The antiretroviral drug distribution to the brain follows the pattern of other drugs' distribution to the brain [124] such that the initial rapid phase in drug distribution reflects the cardiac output and regional blood flow, and the brain, being one of the highly perfused organs in the body, receives most of the drug few minutes after absorption. Subsequent phases of drug distribution are affected by several variables, such as the local cerebral perfusion, lipid solubility of the drug, integrity of tight junctions in the brain, arrangement of the perivascular glial cells, drug binding to plasma protein, and the diffusion gradient [124].

A reduction in resting cerebral blood flow has been demonstrated in HIV patients and linked to development of HAND [125]. Additionally, anaemia is a common haematological disorder among patients with HIV/AIDS [126–128], and when severe, it may compromise cerebral perfusion. Premature atherosclerosis among patients with HIV/AIDS may also adversely affect cerebral perfusion and CNS penetration of antiretroviral drugs [129–132]. On the other hand, inflammation of the brain and the meninges that may complicate HIV infection increases the cerebral blood flow and, potentially, drug access to the CNS.

## 6. Conclusion

Antiretroviral drug concentrations in the CNS reflect interplay of several factors that promote drug entry and others that limit entry. The balance achieved varies between individuals and for each drug. Since manifestations of HAND remain apparent in many patients despite suppression of plasma viremia, optimizing CNS permeability of antiretrovirals should be an integral part of antiretroviral drug development. The ideal agents would have optimal CNS efficacy while being free of neurotoxicity. Research is needed to further understand the effect of antiretroviral CNS penetration on HAND and to discover appropriate interventions.

## Figures and Tables

**Table 1:** Cerebrospinal fluid concentration of some antiretrovirals as a percentage of plasma concentration and their CPE ranking.

Antiretroviral drug	Sample size	CSF concentration as a percentage of plasma concentration (%)		References	Revised CPE rank [22]
		Median (range)	Mean (SD)		
Zidovudine	50	60 (4–262)		[133]	4
	18	2 (0–674)		[134]	
	5		96 (8)	[135]	

Abacavir	54	36		[136]	3
	3^a^	35 (31–44)		[137]	

Lamivudine	55	23 (0–490)		[134]	2

Stavudine	31	20 (0–20.4)		[134]	2

Didanosine	4		27 (14)	[135]	2
	5	Undetected		[134]	

Tenofovir	38	5.7 (3–10)^b^		[84]	1

Efavirenz	69	0.5 (0.26–0.76)^b^		[138]	3
	11	Undetected		[134]	
	26		0.71 (0.37)^c^ in 600 mg	[139]	
	11	1.3 (0.8–1.6)^b^		[140]	

Nevirapine	16	63 (41–77)		[134]	4

Indinavir	19		1.7 (88.6)^c^	[141]	3
	22	16 (0.4–228) or 6 using AUC ratio		[142]	

Lopinavir/r	26	0.23% (0.12–0.75)		[88]	3
	12		0.85 (0.47)	[143]	

Nelfinavir	6	Not detected in CSF		[144]	1
	9	Not detected in CSF		[134]	

Ritonavir	8	0.00 (0.00–52)		[134]	1

Darunavir/r	14		0.5^d^	[145]	3
	14	0.9 (0.3–1.8)		[87]	

Atazanavir	9	1.12 (0.5–13.9)		[146]	2
	8	1.4 (0.6–3.4)^b^		[140]	
	13		3^d^	[145]	

Raltegravir	41	17 (1.8–33.8)^b^		[140]	3
	24	3 (1–61)		[147]	
	35	20.6 (0.5–133)		[148]	

Dolutegravir	12		0.546 (0.480)	[149]	N/A

Maraviroc	7	3.0 (1–10)		[150]	3
	12	2.2 (0.4–17)		[151]	
	12		1.01 (0.29), 4.20 (1.22)^e^	[143]	

^a^Subjects with multiple plasma and CSF samples, ^b^median (IQR), ^c^mean (coefficient of variation), ^d^geometric mean, ^e^% Maraviroc CSF/unbound plasma ratio, and N/A = not available. The ranks assigned to the antiretrovirals in Table 1 are based on revised ranking system proposed by Letendre et al. and not CSF/plasma ratio from the authors findings in the table. Higher CPE score means higher CNS penetration.

**Table 2:** Some reported frequencies of CYP2B6 polymorphism in different African populations.

Population	Frequency (%)	Number of subjects	Reference
African American	20	50	[48]
African American	36	85	[49]
African American	34	93	[152]
Tanzanians	36	95	[153]
Malawians	^∧^31 (15)	26	[60]
Ghanaians	46	42	[152]
Ghanaians	25	800	[78]
Ethiopians	^∧^45.5 (8.7)	262	[45]
Ivory coast	38	41	[152]
Sierra Leone	36	52	[152]
Senegal	60	10	[152]
Guinea	48	21	[152]
West Africa	42	166	[152]
West Africa	50	153	[49]
Yoruba (Ibadan, Nigeria)	35	78	[49]
South Africa	^∧^41 (23)	80	[154]
Xhosa (South Africa)	^∧∧^17	109	[155]
CMA (South Africa)	^∧∧^9	67	[155]
Botswana	36.6	101	[156]
Zimbabwe	49	71	[76]
Uganda	35.6	121	[63]
Uganda	29	7 males	[13]
Mozambique	7	78	[14]

^∧^ *GT (TT) *and ^∧∧^loss of function *CYP2B6* ^*∗*^ *18*.

**Table 3:** Some drugs that are affected by host pharmacogenetics and the resultant effect.

Antiretroviral drug	Enzyme involved	Effect
Efavirenz	CYP2B6	Increase in efavirenz concentrations and increased risk of discontinuation [50–56]
Nevirapine	CYP2B6	Increase in nevirapine plasma concentrations and increase hypersensitivity adverse effect associated with nevirapine [57–60]
Atazanavir	UGT1A1	Hyperbilirubinemia (indirect plasma bilirubin increase) and jaundice [157–160]
Tenofovir	ABCC2, ABCC4	Renal function decline [161–163]
Abacavir	HLA-B^*∗*^5701	Hypersensitivity associated with abacavir [164–167]

**Table 4:** Summary of the factors affecting antiretroviral drug CNS penetrations.

General factors	Pharmacokinetics/pharmacogenetics	Other factors
(i) Blood-Brain barrier (BBB) (ii) Blood-CSF barrier (BCSFB) (iii) Drug transporter	(i) Drug-drug interactions (ii) Drug-herb interactions (iii) Enzyme inhibition/induction (iv) Polymorphism in drug metabolizing enzyme (v) Drug-protein binding (vi) Molecular weight of the antiretroviral drug	(i) Local cerebral blood flow (ii) Presence of chronic inflammation
